# Optimization of parasite DNA enrichment approaches to generate whole genome sequencing data for *Plasmodium falciparum* from low parasitaemia samples

**DOI:** 10.1186/s12936-020-03195-8

**Published:** 2020-03-30

**Authors:** Zalak Shah, Matthew Adams, Kara A. Moser, Biraj Shrestha, Emily M. Stucke, Miriam K. Laufer, David Serre, Joana C. Silva, Shannon Takala-Harrison

**Affiliations:** 1 Malaria Research Program, Center for Vaccine Development and Global Health, University of Maryland School of Medicine, Baltimore, MD USA; 2 Institute for Genome Sciences, University of Maryland School of Medicine, Baltimore, MD USA; 3 Department of Microbiology and Immunology, University of Maryland School of Medicine, Baltimore, MD USA

**Keywords:** *Plasmodium falciparum*, Malaria, Whole genome sequencing, Selective whole genome amplification, Vacuum filtration

## Abstract

**Background:**

Owing to the large amount of host DNA in clinical samples, generation of high-quality *Plasmodium falciparum* whole genome sequencing (WGS) data requires enrichment for parasite DNA. Enrichment is often achieved by leukocyte depletion of infected blood prior to storage. However, leukocyte depletion is difficult in low-resource settings and limits analysis to prospectively-collected samples. As a result, approaches such as selective whole genome amplification (sWGA) are being used to enrich for parasite DNA. However, sWGA has had limited success in generating reliable sequencing data from low parasitaemia samples. In this study, enzymatic digestion with MspJI prior to sWGA and whole genome sequencing was evaluated to determine whether this approach improved genome coverage compared to sWGA alone. The potential of sWGA to cause amplification bias in polyclonal infections was also examined.

**Methods:**

DNA extracted from laboratory-created dried blood spots was treated with a modification-dependent restriction endonuclease, MspJI, and filtered via vacuum filtration. Samples were then selectively amplified using a previously reported sWGA protocol and subjected to WGS. Genome coverage statistics were compared between the optimized sWGA approach and the previously reported sWGA approach performed in parallel. Differential amplification by sWGA was assessed by comparing WGS data generated from lab-created mixtures of parasite isolates, from the same geographical region, generated with or without sWGA.

**Results:**

MspJI digestion did not enrich for parasite DNA. Samples that underwent vacuum filtration (without MspJI digestion) prior to sWGA had the highest parasite DNA concentration and displayed greater genome coverage compared to MspJI + sWGA and sWGA alone, particularly for low parasitaemia samples. The optimized sWGA (filtration + sWGA) approach was successfully used to generate WGS data from 218 non-leukocyte depleted field samples from Malawi. Sequences from lab-created mixtures of parasites did not show evidence of differential amplification of parasite strains compared to directly sequenced samples.

**Conclusion:**

This optimized sWGA approach is a reliable method to obtain WGS data from non-leukocyte depleted, low parasitaemia samples. The absence of amplification bias in data generated from mixtures of isolates from the same geographic region suggests that this approach can be appropriately used for molecular epidemiological studies.

## Background

Next-generation sequencing has greatly advanced research on malaria parasite genomics. Several molecular epidemiological studies have used genomic approaches in an effort to better understand *Plasmodium falciparum* genetic diversity in relation to malaria transmission, drug resistance and vaccine design [1–5]. However, a majority of such population genomics studies rely on whole genome sequencing of samples collected in malaria endemic areas. Since patient blood samples contain mostly human DNA, enrichment for parasite DNA is required in order to obtain parasite sequence data with adequate genome coverage. Leukocyte depletion is an effective method for reducing the amount of host DNA for parasite sequencing and hence increase the proportion of parasite DNA prior to sequencing; however, depletion must be performed within hours of sample collection and can be logistically challenging in some resource-limited settings [6, 7]. In addition, once the sample is frozen and cells are lysed, leukocyte depletion is no longer effective, thus limiting the application of this approach to prospectively-collected samples. To reduce the need for extensive sample processing in the field and to enable examination of the wealth of historical samples collected as dried blood spots or whole venous blood, malaria researchers have explored alternative parasite DNA enrichment approaches including enzymatic digestion of human DNA [8], selective whole genome amplification (sWGA) [9], and hybrid selection (capture-based method) [10].

MspJI is a restriction endonuclease that cleaves specific motifs containing methylated cytosines that has been used to selectively digest human DNA prior to parasite DNA sequencing [11]. The success of this approach is based on the assumption of different methylation patterns in the human and parasite genomes; however, methylation patterns in *P. falciparum* are not fully understood [12]. sWGA of the parasite genome over the human genome has also shown promising results as a method for enrichment of parasite DNA prior to whole genome sequencing. This approach uses multiple displacement amplification with phi29 DNA polymerase using primers binding at greater density in the parasite genome compared to the human genome. Phi29 results in amplification of long DNA fragments and is known to have a low error rate. An existing sWGA protocol has been shown to work best with samples that have parasitaemia greater than ~ 1200 parasites/µL [9]. While this parasitaemia threshold may allow sequencing of most clinical infections, it limits studies of lower parasitaemia infections, including submicroscopic infections that may significantly contribute to the malaria burden in some areas [13]. In addition, because sWGA primers were designed against the 3D7 reference sequence without consideration of *P. falciparum* genetic diversity, the potential for differential amplification of particular parasite clones within a polyclonal infection (e.g. those most genetically similar to the reference) is a concern. Since a large proportion of infections from high-transmission areas are polyclonal, the possibility of amplification bias introduced by sWGA warrants further investigation, as such bias could lead to inaccurate inferences in downstream analyses.

In this study, enzymatic digestion with MspJI prior to sWGA and whole genome sequencing was evaluated to determine whether this approach improved genome coverage compared to sWGA alone when applied to samples representing a range of parasitaemias. In addition, this study also evaluated whether the optimized sWGA protocol results in biased estimates of multiplicity of infection or allele frequencies by comparing whole genome sequence data generated from laboratory-created mixtures of isolates from Malawi that underwent sWGA prior to sequencing or were directly sequenced.

## Methods

### Laboratory-created samples to evaluate enrichment approaches

#### Dried blood spots

To test the different parasite DNA enrichment approaches, dried blood spots were created by mixing cultured NF54-infected red blood cells with uninfected whole human blood. The laboratory-adapted isolate, NF54, was maintained in culture following the method of Trager and Jensen [14]. Parasite concentrations were microscopically enumerated from sorbitol-synchronized ring-stage NF54 culture and mixed with uninfected whole human blood (Interstate Blood Bank, Nashville, TN) resulting in parasite concentrations ranging from 10,000 to 500 parasites/µL and subsequently spotting 12.5 μL of blood onto Whatman 3MM filter paper. DNA was extracted from dried blood spots using the protocol described by Zainabadi et al. [15]. The DNA was treated under three conditions, including sWGA only and MspJI or MspJI^−^ control (no enzyme) + followed by sWGA, as illustrated in Additional file 1: Figure S1. The MpsJI^−^ condition followed the same protocol as MspJI, without the enzyme.

#### Mixtures of DNA to assess amplification bias

DNA from four previously cultured and sequenced field isolates from Malawi [16] were mixed in equal proportions to assess potential amplification bias introduced by sWGA. The DNA concentration for each sample was measured using a picogreen assay (Thermo Fisher Scientific, Waltham, MA) and the samples were mixed in equal proportions. None of the cultured isolates represented polyclonal infections, based on analysis of prior sequencing data using estMOI [17]. The sample was further split into six tubes, three of which underwent direct whole genome sequencing and the other three which underwent sWGA, followed by whole genome sequencing, as shown in Fig. 4.

#### MspJI digestion

MspJI digestion was performed in a 0.2 mL 96-well PCR plate. The reaction mixture contained 1X CutSmart Buffer, 10 μg of bovine serum albumin and 6 units of MspJI (New England Biolabs, Ipswich, MA). The MspJI digestion control (i.e., MspJI^−^) contained the same buffers but excluded the enzyme. 25 µL of sample DNA was added to both reaction mixtures (total volume, 30 µL), and reactions were incubated in a thermocycler. Two different incubation protocols were tested, one with a 16 h incubation at 37 °C, followed heating at 65 °C for 20 min to inactivate the enzyme and cooling at 4 °C, and a second protocol where the 37 °C incubation lasted only 4 h.

#### Vacuum filtration of DNA

Following enzymatic digestion, the entire reaction mixture was transferred to a MultiScreen^®^ PCR Filter Plate (Millipore) and filtered to remove digested DNA fragments using a MultiScreen^®^ Vacuum Manifold with a pressure of − 7 inches Hg until the wells were emptied and the filters appeared dry. Filtered samples were reconstituted with 30 µL of water, and the plate was gently agitated for 15 min. Samples were then transferred to a new plate.

#### sWGA

Amplification was performed in a 0.2 mL 96-well PCR plate. The reaction mixture contained 1X BSA, 1 mM dNTPs, 2.5 µM of each amplification primer, 1X Phi29 reaction buffer and 30 units of Phi29 polymerase. 17 µL of template DNA was added to the reaction mixture (total volume, 50 μL) which was then placed in a thermocycler programmed for a stepdown protocol (35 °C for 5 min, 34 °C for 10 min, 33 °C for 15 min, 32 °C for 20 min, 31 °C for 30 min, 30 °C for 16 h), followed by heating at 65 °C to inactivate the enzyme and cooling at 4 °C. Primers used for sWGA were the same as those published by Oyola et al. [9] (see Additional file 1).

#### qPCR

Quantitative PCR of the human actin gene and *P. falciparum* 18S rRNA gene was used to estimate the amount of human and parasite DNA, respectively, before and after sWGA. The reaction mixture contained QuantiTech 2X QT Multiplex Master Mix, 10 µM of each of the primers and 1.5 μL of template DNA (total volume, 10 μL). A two-tailed Mann–Whitney U test was used to estimate differences between different experimental conditions (e.g. MspJI-sWGA, MspJI^−^-sWGA, sWGA).

#### Whole genome sequencing

Genomic DNA libraries were constructed for sequencing using the KAPA Library Preparation Kit (Kapa Biosystems, Woburn, MA). DNA (500 ηg) was fragmented with the Covaris E210 to ~ 200 bp. Libraries were prepared using a modified version of the manufacturer’s protocol. The DNA was purified between enzymatic reactions and library size selection was performed with AMPure XT beads. Libraries were assessed for concentration and fragment size using the DNA High Sensitivity Assay on the LabChip GX (Perkin Elmer, Waltham, MA). Library concentrations were also assessed by qPCR using the KAPA Library Quantification Kit. Libraries were pooled and sequenced on a 150 bp paired-end Illumina HiSeq 4000 run (Illumina, San Diego, CA).

### Data analysis

#### Read mapping and coverage

Each dataset was analysed by mapping raw fastq files to the 3D7 reference genome using Bowtie2 [18]. Bam files were processed according to GATK’s Best Practices workflow to obtain analysis-ready reads [19, 20]. Bedtools [21] was used to generate coverage and depth estimates from the processed reads. Differences in the proportion of the genome covered in samples from untreated vs. filtered sWGA were tested using the z-score test for difference in proportions.

#### Variant calling

GATK’s Best Practices workflow was followed for variant calling [19, 20]. Haplotype Caller was used in reference confidence mode to create genomic variant call format (GVCF) files for each sample and joint SNP Calling (GATK v3.7). Variants were removed if they met the following filtering criteria: QD < 2.0, FS > 60.0, MQ < 40.0, MQRankSum < − 12.5, ReadPosRankSum < − 8.0, QUAL < 50. Variant sites with > 20% missing genotypes were additionally removed using vcftools.

#### Assessment of amplification bias

Whole genome sequence data generated from laboratory-created isolate mixtures that either underwent sWGA and sequencing or direct sequencing were compared to evaluate the potential for sWGA to introduce amplification bias. The experimental design is illustrated in Fig. 4. Reference allele frequencies for each sample were estimated using samtools mpileup and were compared to examine the variability between and within samples from sWGA and non-sWGA groups. Sites with coverage depth lower than 20X were excluded from this analysis, as were sites not called in all samples. After applying these filters, 1,786,088 sites remained. The distribution and Spearman correlation coefficient (rho) were estimated in R.

*F*_WS_, a measure of within-sample diversity, was used to estimate infection complexity for each isolate mixture for comparison between the sWGA and non-sWGA conditions. *F*_WS_ was estimated using the R package, *moimix* (https://github.com/bahlolab/moimix). Significance was determined using the Mann–Whitney U test. Only the core *P. falciparum* genome was used to estimate *F*_WS_ [22].

The composition of each laboratory-created mixture was also compared to determine if there were significant differences between the sWGA and non-sWGA groups, implying differential amplification of certain strains over others. To assess potential amplification bias, the proportion of isolate-specific SNPs called from WGS data generated from isolate mixtures that did or did not undergo sWGA prior to sequencing was compared for each isolate. Isolate-specific SNPs were identified by comparing WGS data from each of the four isolates used to create the experimental mixtures. All variant sites with missing alleles in any of the isolates were removed from the analysis. For each isolate mixture, the predominant allele (defined as an allele comprising > 70% of reads) at each position was called; if no allele was predominant based on this threshold, the SNP was called missing. The positions of unique SNPs for each isolate were extracted from the sequence data generated from the isolate mixtures. The proportion of unique SNPs from each isolate in the mixture was estimated and compared between the sWGA and non-sWGA groups. Significance was estimated using a z-score test for difference in proportions.

## Results

### Vacuum filtration, but not enzyme digestion, prior to sWGA increases parasite DNA concentration and improves the quality of whole genome sequence data

**Fig. 1:**
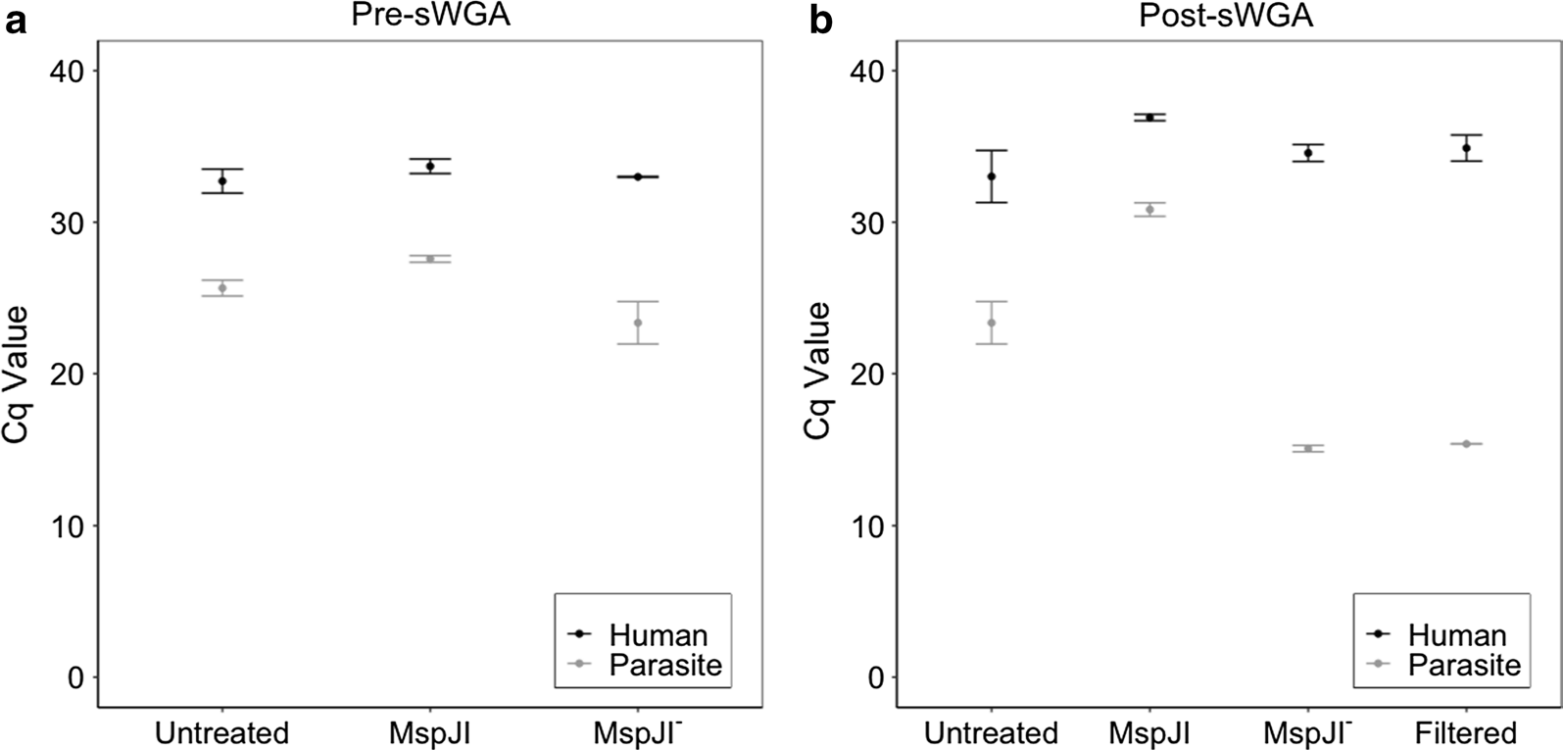
Effect of MspJI and sWGA treatments on parasite DNA concentration. **a** Human and *P. falciparum* Cq values prior to sWGA on samples with 10,000 parasites/μL (n = 3). Cq value indicates the number of cycles required to detect a signal, where higher Cq values indicate lower DNA concentrations. **b** Human and *P. falciparum* Cq values after sWGA on samples with 10,000 parasites/μL (n = 3)

Laboratory-created dried blood spots representing a range of parasitaemias were created to test the different DNA enrichment approaches. DNA extracted from the dried blood spots underwent one of three conditions prior to sWGA: (1) MspJI digestion, (2) MspJI^−^ control (same conditions as MspJI but without enzyme), or (3) untreated, as illustrated in Additional file 1: Figure S1. Samples that underwent MspJI digestion had significantly less human (p = 0.028, Mann–Whitney U test) and parasite DNA (p = 0.028, Mann–Whitney U test) compared to the untreated samples. Similarly, MspJI-digested samples also had significantly less human (p = 0.028, Mann–Whitney U test) and parasite DNA (p = 0.028, Mann–Whitney U test) compared to the MspJI^−^ control (Fig. 1a). This pattern was consistent following sWGA, with MspJI-sWGA samples having significantly less human DNA (p-value = 0.028, Mann–Whitney U test) as well as parasite DNA (p-value = 0.028, Mann–Whitney U test) compared to the untreated-sWGA samples. Surprisingly, the MspJI^−^ control samples that underwent sWGA had a significantly higher parasite DNA concentration compared to samples digested with MspJI (p-value = 0.028, Mann–Whitney U test) and untreated samples (p-value = 0.028, Mann–Whitney U test) that underwent sWGA. Further experiments suggested that the vacuum filtration step in the MspJI^−^ condition (Additional file 1: Figure S1) was likely responsible for the improved parasite DNA concentration (Fig. 1b, p-value = 0.028, Mann–Whitney U test), possibly due to removal of small DNA fragments that may lead to non-specific binding. This result was also consistent across samples with lower parasitaemias, ranging from 5000 to 500 parasites/µL (Additional file 1: Figure S2, p-value = 0.028, Mann–Whitney U test).

**Fig. 2:**
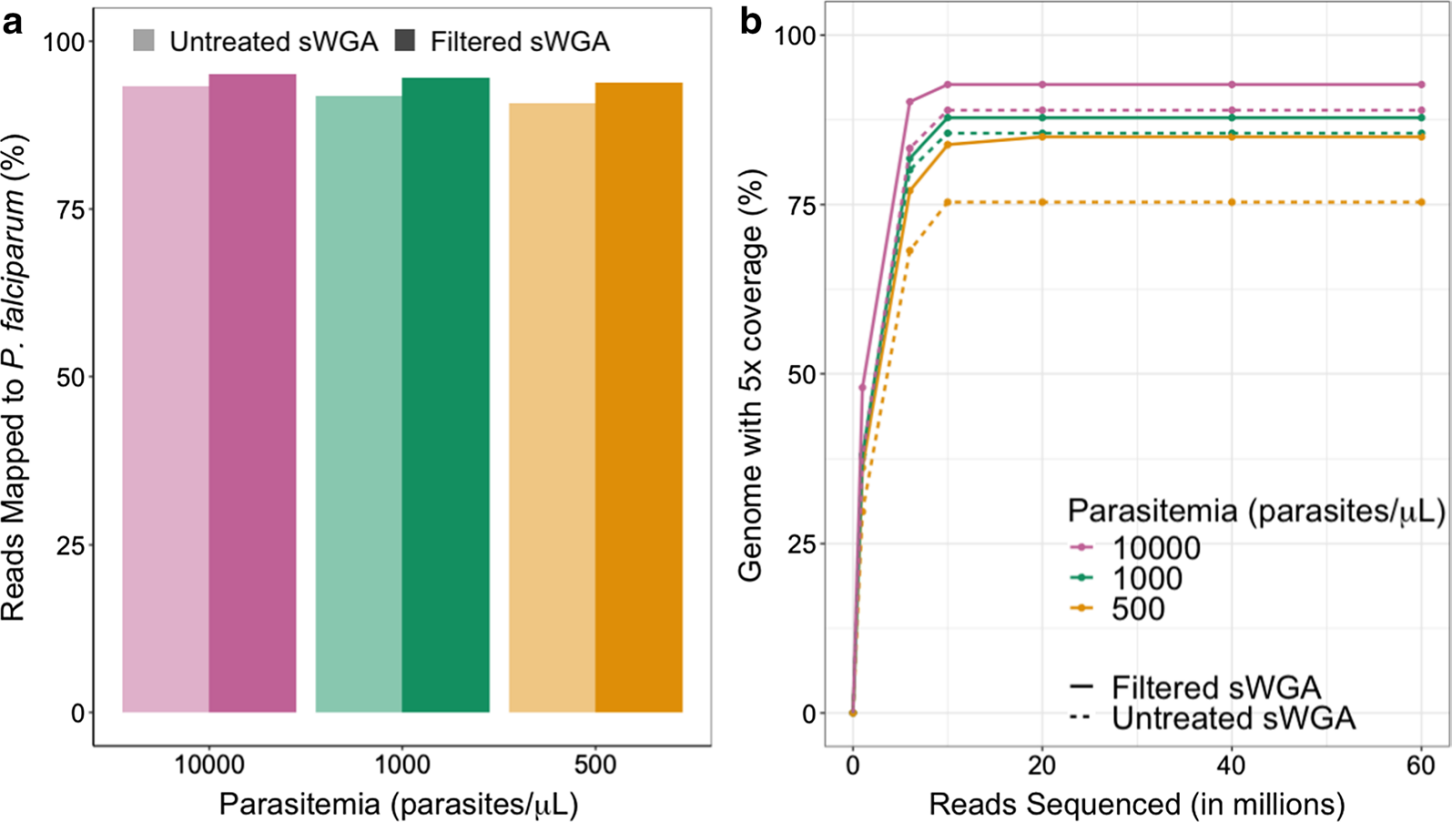
*Plasmodium falciparum* genome coverage in filtered and untreated samples that underwent sWGA. **a** The percentage of reads that mapped to the *P. falciparum* 3D7 reference are shown for filtered and untreated samples with different parasitaemias that underwent sWGA prior to sequencing. **b** Percentage of the *P. falciparum* 3D7 genome with at least 5X coverage is shown relative to the number of reads sequenced (in millions) in filtered and untreated samples of different parasitaemias that underwent sWGA prior to sequencing

The untreated-sWGA (no treatment, only sWGA) and filtered-sWGA samples (vacuum filtration, followed by sWGA), with different parasitaemias (10,000, 1000, 500 parasites/µL), underwent whole genome sequencing, and the sequence reads were trimmed and mapped to the *P. falciparum* 3D7 reference genome. As seen in Fig. 2a, the filtered-sWGA samples had a higher percentage of reads map to 3D7 (p < 0.0001, z score test for difference in proportions). This pattern was more notable for samples with lower parasitaemia (Fig. 2a). Sequence data from filtered-sWGA samples displayed a slightly higher percent of the genome with 5X coverage per million reads sequenced compared to sequence data from untreated-sWGA samples (Fig. 2b). Further in-depth coverage analysis showed that this pattern was consistent across all chromosomes (Additional file 1: Figure S3A). Visual inspection of coverage along chromosome 1 showed uneven coverage in both filtered and unfiltered samples that underwent sWGA but filtered-sWGA samples had higher coverage in most regions, particularly in the lowest parasitaemia sample. Indeed, in the lowest parasitaemia sample, higher coverage was observed in several regions where the untreated-sWGA sample had very little or no coverage (Additional file 1: Figure S3B).

**Fig. 3:**
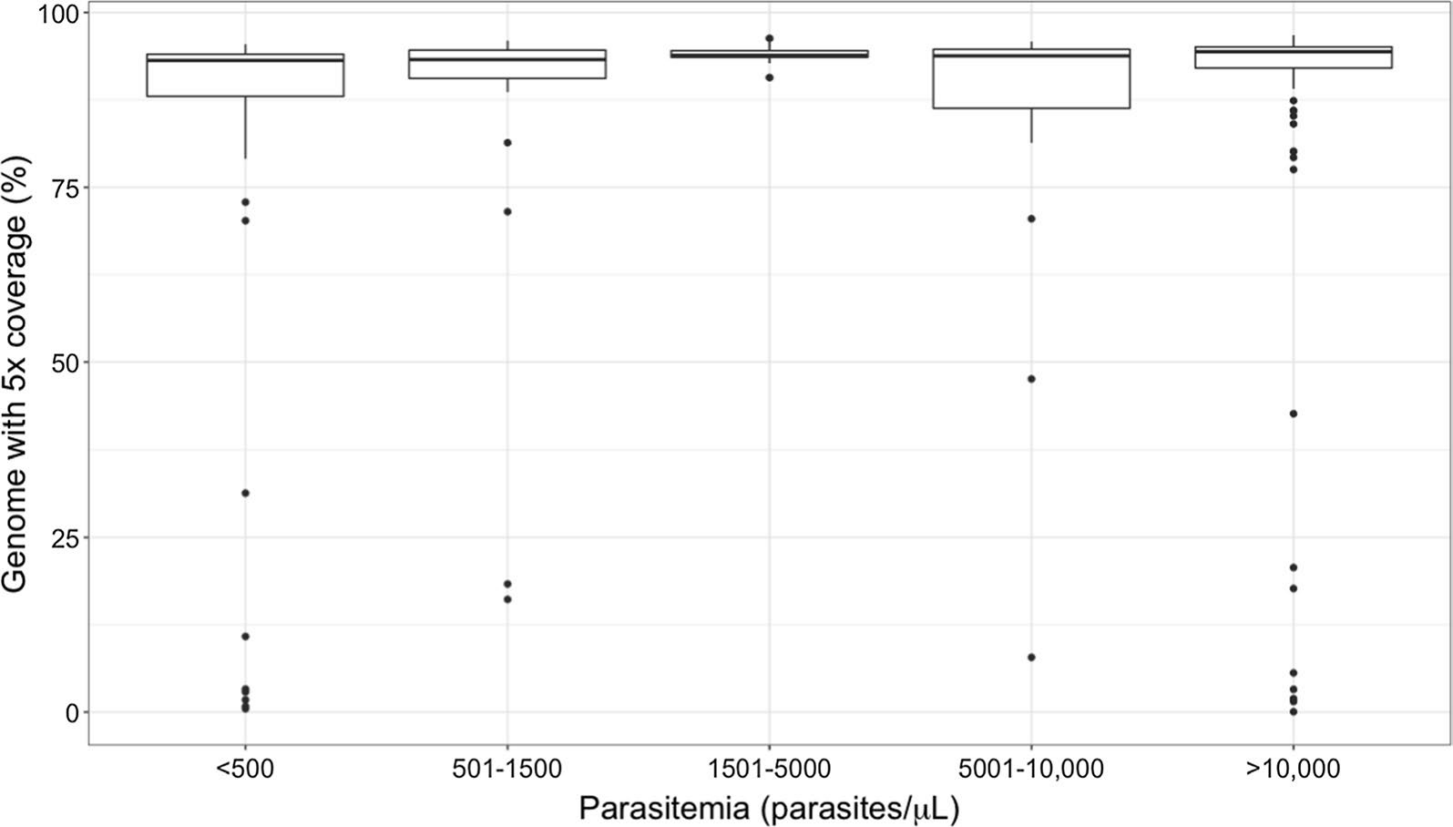
Percent of genome with 5X coverage in whole genome sequences of field isolates with different parasitaemias

Next, the optimized sWGA protocol was tested on 218 red blood cell pellets from Malawi that were PCR-positive for *P. falciparum* and had parasitaemias, ranging from 0 to > 200,000 parasites/µL (by microscopy). Only 23/218 (10.55%) samples had less than 75% of the genome with ≥ 5X coverage. Samples with > 75% of the genome with ≥ 5X coverage had a median average read depth of ~ 137X. Out of 50 samples with parasitaemia < 500 parasites/mL, only 9 (18%) samples had less than 75% of the genome with ≥ 5X coverage, while the remaining samples with > 75% of the genome with ≥ 5X coverage had a median average read depth of ~ 129X. The correlation between percent genome coverage and parasitaemia was also estimated (Fig. 3) and found to be weak, but statistically significant (p = 0.00012, Spearman’s correlation rho = 0.25).

### Optimized sWGA does not show evidence of amplification bias when applied to mixtures of parasite isolates from the same geographic region

**Fig. 4:**
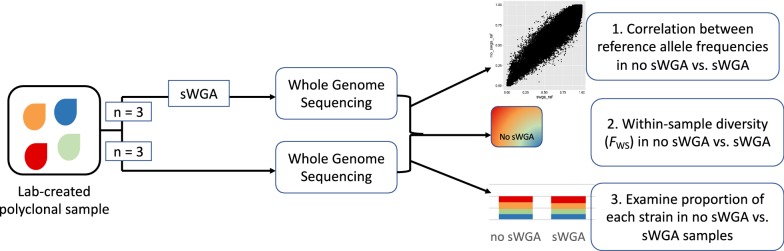
Schematic of experimental design to evaluate potential amplification bias in samples undergoing sWGA

To examine the potential for amplification bias introduced by sWGA, equal mixtures of four *P. falciparum* isolates from Malawi were created. Three aliquots of these mixtures underwent sWGA followed by whole genome sequencing and three underwent whole genome sequencing without prior sWGA (Fig. 4). Mixtures that underwent sWGA had a larger proportion of sequenced reads that mapped to the 3D7 reference genome compared to directly-sequenced mixtures, while directly-sequenced mixtures had a higher percentage of the genome with at least 5X coverage (Table 1).

**Table 1:** Sequencing statistics in samples that underwent direct sequencing versus sWGA

	Direct Sequencing (n = 3)	sWGA (n = 3)
Total reads sequenced	30,878,334	33,586,527
Reads mapped to *P. falciparum* (%)	92.21	95.52
Genome with 5X coverage (%)	98.68	95.53
Mean coverage depth	196X	213X
Total SNPs	32,775	22,355
*F*_WS_	0.068 ± 0.009	0.116 ± 0.015

Three approaches were used to evaluate the potential for amplification bias by sWGA (Fig. 4). First, the correlation between reference allele frequencies was estimated for each variant site in sequence data generated from mixtures that did or did not undergo sWGA prior to sequencing. Based on visual examination, the distribution of reference allele frequencies was similar in all six samples, but directly-sequenced samples had more clearly distinct peaks representing each of the four isolates compared to samples that underwent sWGA prior to sequencing (Additional file 1: Figure S4). The correlation between reference allele frequencies was high (> 94%) and similar within and between sWGA or directly-sequenced samples (Additional file 1: Figure S5).

Second, *F*_ws_, a measure of within-sample diversity [2, 23, 24], was estimated to examine differences in infection complexity between samples that underwent sWGA prior to sequencing and those that did not. *F*_ws_ values range from 0 to 1, with 0 indicating a balanced mixture of highly unrelated clones and 1 indicating a single clone. Directly-sequenced samples had lower average *F*_ws_ estimates than samples that underwent sWGA (Table 1). However, when *F*_ws_ was estimated based on the subset of SNPs called in both groups, there was no significant difference in *F*_ws_ between groups (p-value = 0.1, Mann–Whitney U Test).

**Fig. 5:**
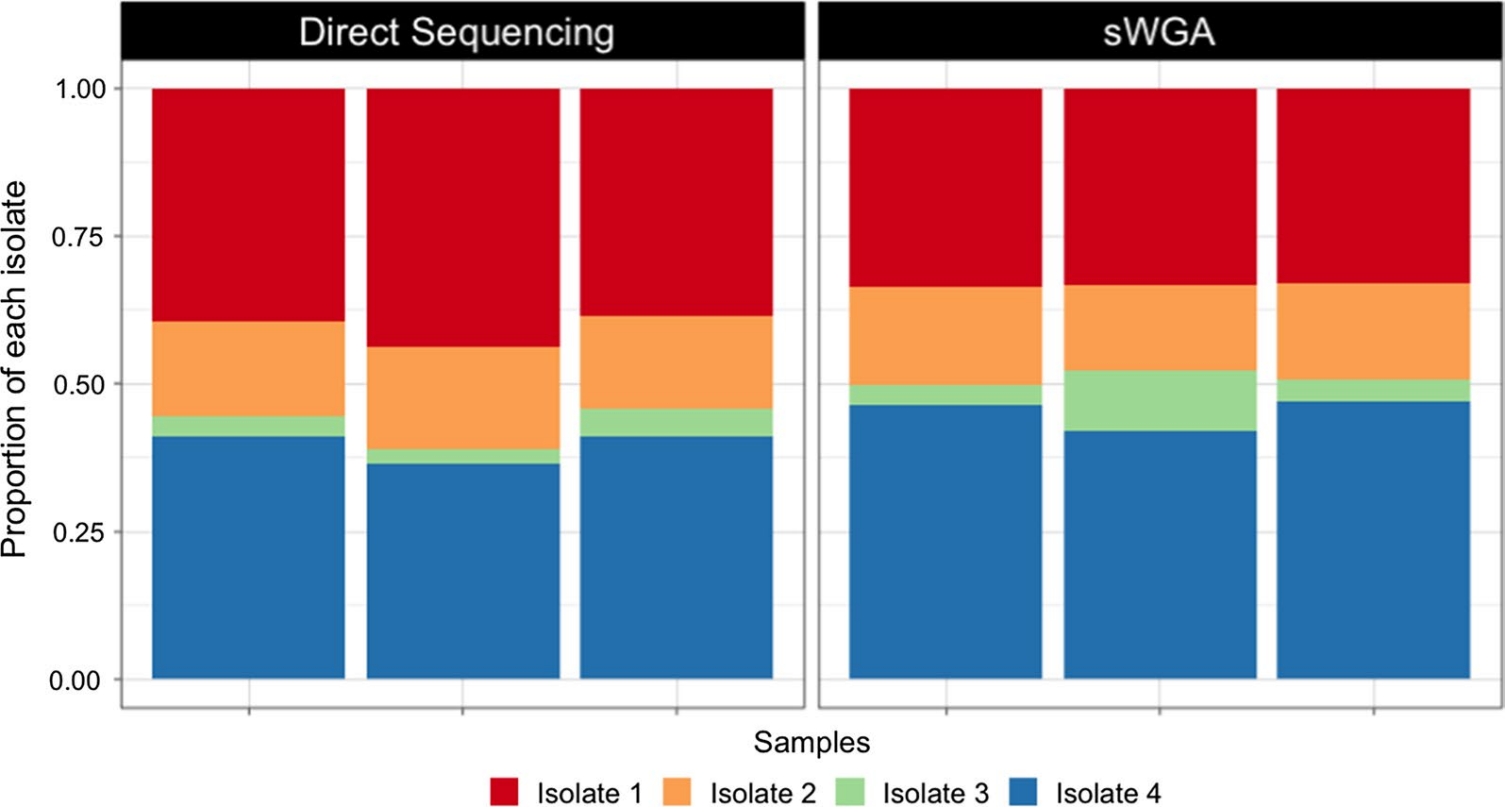
Proportion of isolate-specific SNPs in mixtures that underwent sWGA prior to sequencing or were directly sequenced

Finally, the proportion of each isolate in our isolate mixtures was compared based on the frequency of isolate-specific variants in sequence data generated from the mixtures that did or did not undergo sWGA. The proportion of isolate-specific SNPs in each mixture did not differ significantly between mixtures that underwent sWGA and those that did not (Fig. 5).

## Discussion

While advances in next-generation sequencing technologies have greatly expanded research in the field of malaria genomics, the difficulties of enriching for malaria parasite DNA in clinical samples, particularly those collected from submicroscopic infections, has limited population genomics analyses to include mostly high-parasitaemia, symptomatic infections. In this study, two published parasite DNA enrichment approaches were combined, namely enzyme digestion of human DNA [8] and sWGA [9], to determine whether the combined approaches improved the ability to generate high-quality whole genome sequence data from non-leukocyte-depleted clinical samples with low parasitaemia. Oyola et al. reported up to ~ 9-fold *P. falciparum* DNA enrichment resulting in > 98% of the parasite genome with at least 5X coverage when using MspJI digestion only (no sWGA) prior to sequencing [8]. However, in this study, enzyme digestion with MspJI resulted in a significant decrease in both human and parasite DNA concentrations, before and after sWGA. This reduced DNA concentration may be due to digestion of both human and parasite DNA, consistent with the presence of a “smear” of small DNA fragments observed when digested samples were subjected to gel electrophoresis (data not shown). This finding contrasts with that of Oyola et al. who observed a band representing intact parasite DNA along with digested human DNA following MspJI digestion, although it is notable that the amount of starting DNA (1 µg total) used in the Oyola study was much larger than that obtained from the dried blood spots in this study, and likely larger than what would be obtained from dried blood spots collected in the field. In addition, Cowell et al. [24] observed no significant difference in genome coverage with digestion using MspJI or FspEI prior to sWGA and sequencing of *Plasmodium vivax*. However, since MspJI targets specific motifs containing methylated cytosines, and *P. vivax* has higher GC content than *P. falciparum*, it is possible that the targeted motifs may be more common in *P. vivax*, leading to the failure of MspJI in this context. Though MspJI digestion was not successful, further investigation is warranted to identify alternative enzymes that target the human genome over the parasite genome that could potentially perform better and be useful in combination with sWGA.

While digestion with MpsJI did not improve parasite DNA concentration, surprisingly, the parasite DNA concentration was significantly greater in the MspJI^−^ control compared to MspJI and no treatment. Subsequent experiments suggested that this increased DNA concentration resulted from the vacuum filtration step. Indeed, it was discovered that the filtration of extracted DNA prior to sWGA resulted in a greater parasite DNA concentration compared to unfiltered DNA that underwent sWGA. This result may be due to removal of small fragments of parasite DNA that may bind sWGA primers but not lead to effective amplification because of their short length. The increased DNA concentration obtained from the optimized sWGA protocol also resulted in increased genome coverage of whole genome sequencing data, although this increase was most pronounced in lower parasitaemia samples.

Using the optimized sWGA approach, high coverage whole genome sequencing data was obtained from DNA extracted from laboratory-created dried blood spots with parasitaemias as low as 500 parasites/µL, as well as from non-leukocyte depleted red blood cell pellets from the field, including 41 samples from low parasitaemia, sub-microscopic infections. Although statistically significant, the correlation between parasitaemia and percent of the genome with 5X coverage was weak, suggesting that this optimized sWGA approach can be used to obtain high-quality whole genome sequence data from low parasitaemia samples. Additional testing of this approach on dried blood spots stored under different environmental conditions and storage times will be required to further characterize which samples are likely to yield successful results using this optimized approach. If the parasite DNA is highly degraded, this optimized approach may not be successful, both because small DNA fragments may be removed during filtration and lead to less effective whole genome amplification and because of a general lack of longer fragments needed for successful sWGA. For more highly degraded samples, capture-based methods of enrichment may yield better results.

Amplification bias following sWGA or capture-based parasite DNA enrichment has been largely understudied but is essential to understand given the high prevalence of polyclonal infections in high transmission areas. The potential for amplification bias following sWGA was further explored by comparing whole genome sequence data generated from experimental mixtures of parasite DNA from four culture-adapted parasite isolates from Malawi that were either directly sequenced or underwent sWGA prior to sequencing. Consistent with other studies, genome coverage of sequence data generated from samples that underwent sWGA was more uneven compared to coverage of sequence data generated through direct sequencing, most likely due to the sparse sWGA primer coverage in diverse and AT-rich subtelomeric regions [9, 25]. However, per base reference allele frequencies within the core genome were highly correlated both within and between the sWGA and directly-sequenced groups, suggesting that sWGA is not substantially biasing allele frequencies. Experimental mixtures that underwent sWGA had significantly higher estimates of *F*_ws_, implying some loss of within-mixture diversity. This result is in agreement with the results of Cowell et al. [24] who found reduced estimates of infection complexity following sWGA of *P. vivax* DNA, based on analysis of both sequence data and microsatellites, and may be explained by differences in genome coverage between groups. Indeed, directly sequenced samples had higher genome coverage than samples that underwent sWGA, even in the core genome. To test this hypothesis, *F*_WS_ was estimated based only on SNPs called in both the sWGA samples and the directly sequenced samples and no significant difference in *F*_WS_ was observed between groups, suggesting the lower diversity in samples that underwent sWGA was not the result of amplification bias favouring some isolates over others. This conclusion is also supported by the lack of significant differences in the proportion of isolate-specific variants between the sWGA and directly sequenced isolates. More in-depth analysis will be required to evaluate which genomic regions have reduced coverage in sWGA samples and the implications for downstream analyses.

While preferential amplification was not observed based on equal mixtures of isolates from Malawi based on variants called against the 3D7 reference (believed to be of African origin [16, 26]), it would be informative to evaluate this phenomenon in mixtures of parasites from other geographic regions or in varying proportions to determine whether one set of sWGA primers will provide unbiased results and high genome coverage in all settings or whether sWGA primers designed based on regional reference genomes is necessary. Because parasites from different continents are more genetically differentiated than parasites from the same geographic region [2], amplification bias may be more of a concern for comparisons involving parasites sampled from different continents than for comparisons of parasites from the same geographic region.

## Conclusions

The optimized sWGA approach is a reliable method to obtain WGS data from non-leukocyte depleted, low parasitaemia samples. The absence of amplification bias in data generated from mixtures of isolates from the same geographic region suggests that this approach can be appropriately used for molecular epidemiological studies.

## Supplementary information

**Additional file 1.** Supplementary material.

## Data Availability

The datasets used and/or analyzed during the current study are available from the corresponding author on reasonable request.

## Abbreviations

DBS: Dried blood spots
MOI: Multiplicity of infection
sWGA: Selective whole genome amplification
WGS: Whole genome sequencing
