# Supplementary material for: Optimization of parasite DNA enrichment approaches to generate whole genome sequencing data for Plasmodium falciparum from low parasitaemia samples
Source: Malar J. 2020 Mar 30;19:135. doi: 10.1186/s12936-020-03195-8 (PMC7106660; doi:10.1186/s12936-020-03195-8)
Supplement: Supplementary file 1 — Additional file 1. Supplementary material. [file 12936_2020_3195_MOESM1_ESM.pdf]

Supplementary Figures

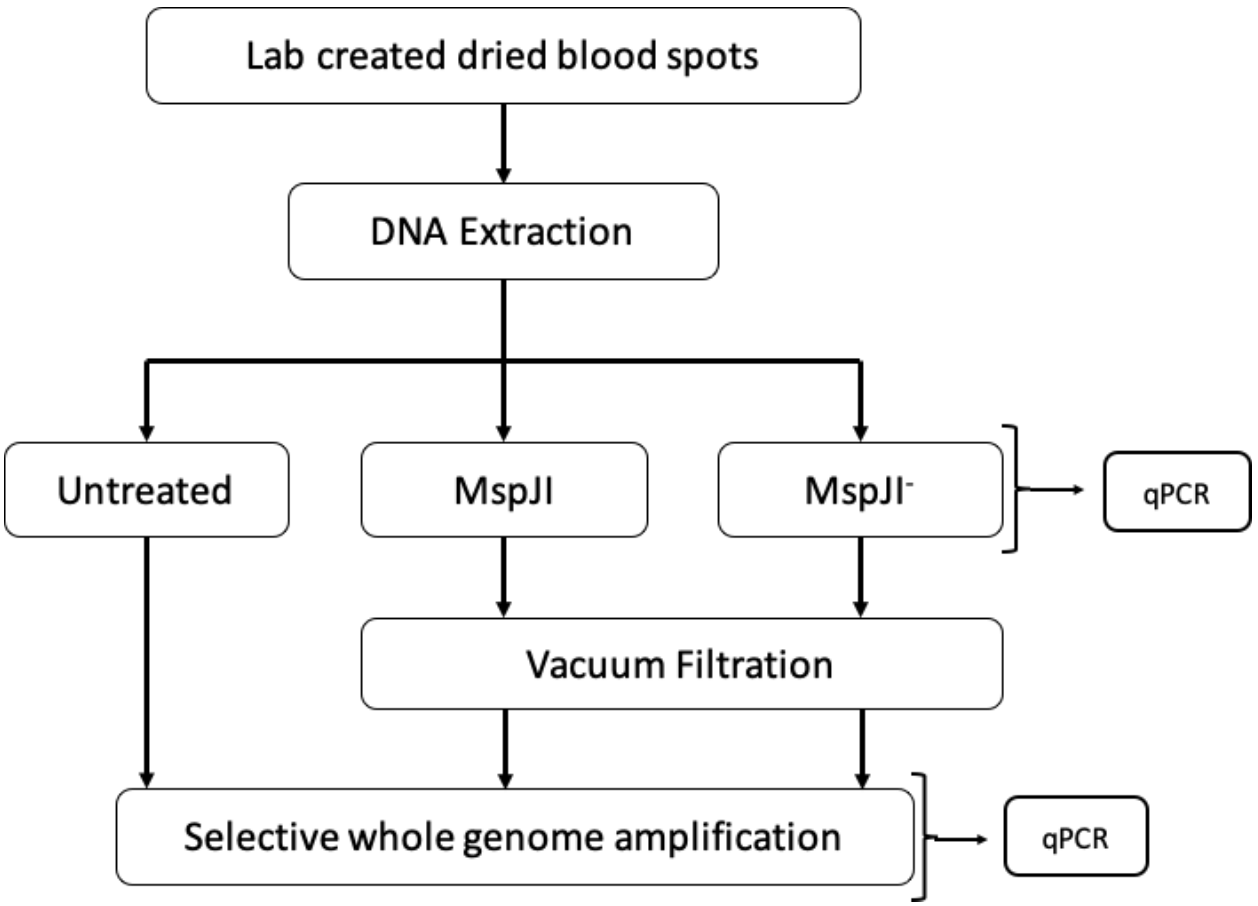

Additional file: Figure S1. Flowchart describing the experimental design to optimize selective whole genome amplification. Vacuum Filtration was done using MultiScreen® Filter Plate (Millipore).

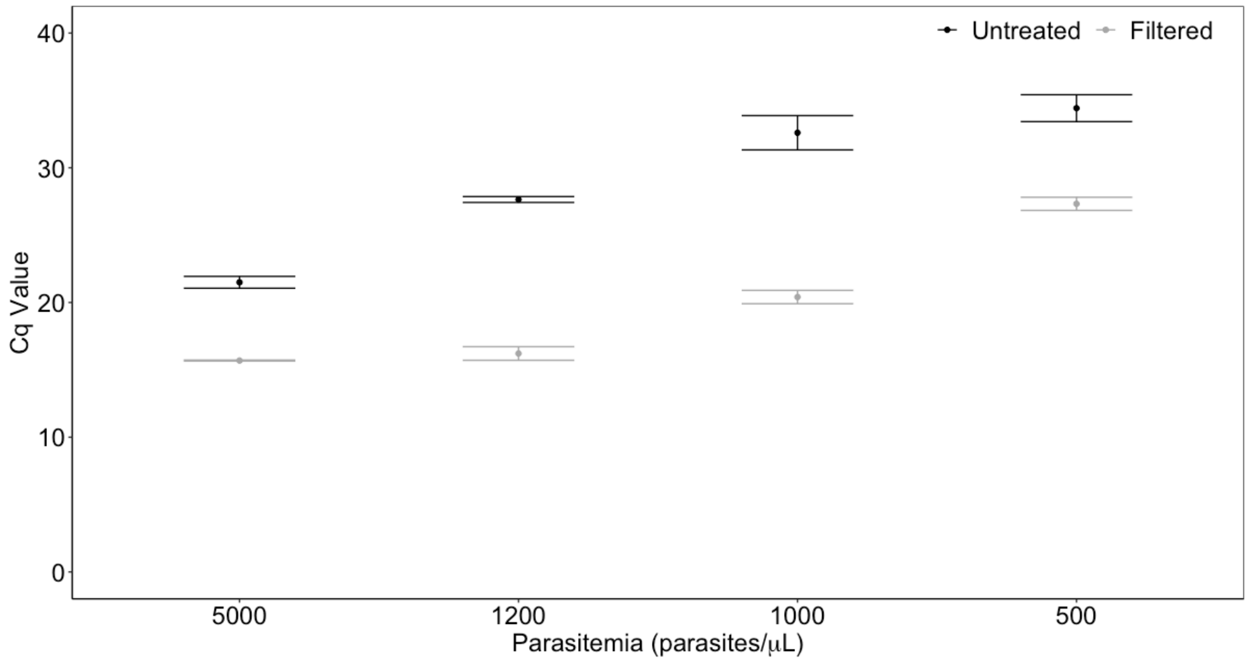

Additional file: Figure S2: Effect of filtering on parasite DNA concentration in parasites with lower parasitemias (n = 3). *P. falciparum* Cq values for untreated-sWGA and filtered-sWGA condition in samples with lower parasitemias. Error bars represent standard error.

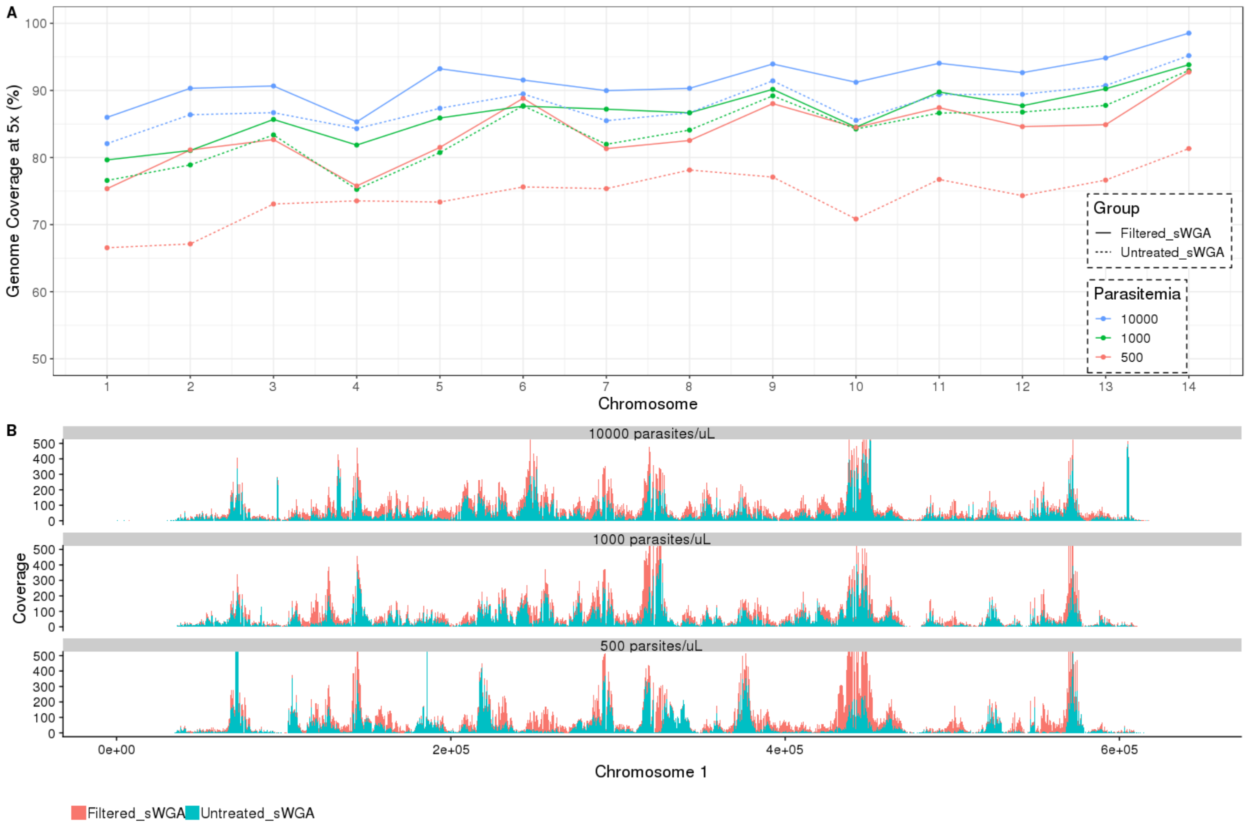

Additional file: Figure S3. A) Average coverage per chromosome in untreated-sWGA *vs*. filtered-sWGA samples. B) Coverage over Chromosome 1.

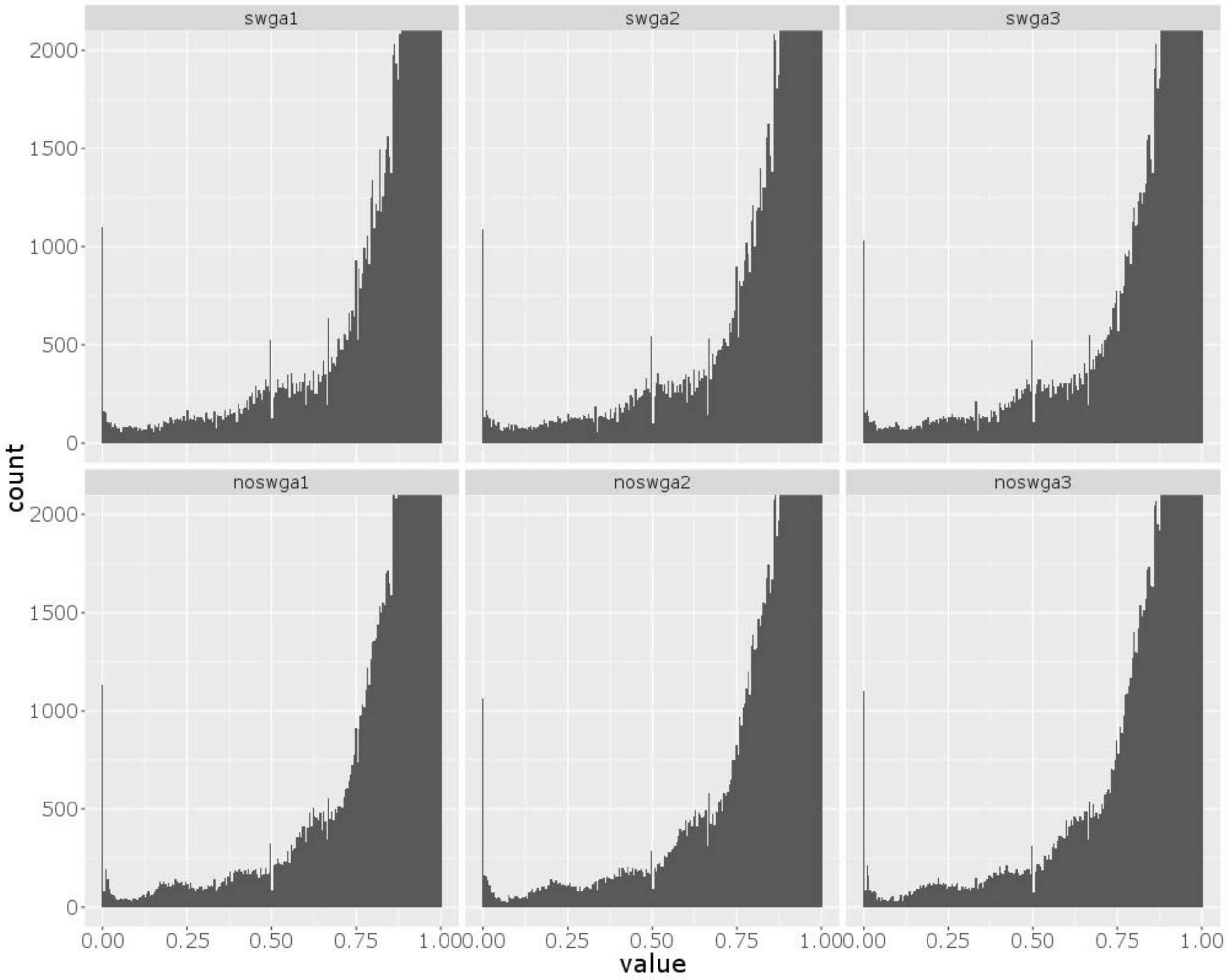

Additional file: Figure S4. Distribution of reference allele frequencies for each site in the genome for all mixed samples. The y-axis shows the number of variable positions for proportion of reads carrying the 3D7 reference allele shown on the x-axis.

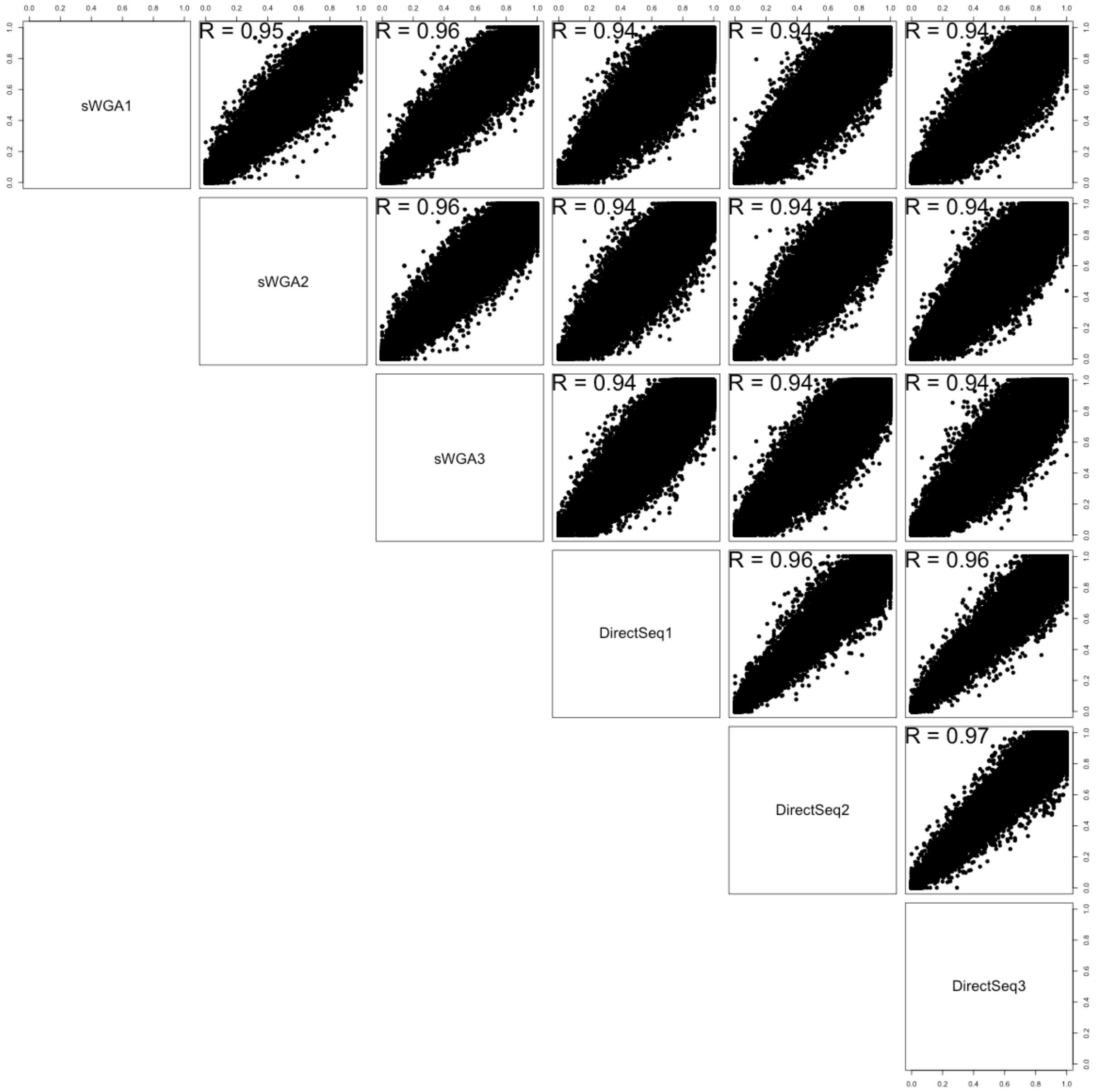

Additional file: Figure S5. Correlation between reference allele frequency estimates of all mixed samples. The plots show correlation between samples from within each group and also between both the groups (sWGA and no sWGA). The upper left side of the matrix shows the corresponding correlation coefficient r^2 for each comparison.

Supplementary Methods

DNA Extraction

DNA was extracted using the New Extraction Method (Zainabadi *et al*) protocol (from blood) and elute with 50 μL TE buffer.

Filtration

25 μL DNA was diluted with 5 μL water. The diluted DNA was then filtered using Millipore vacuum filtration plate and elute with 30 μL water, followed by gentle agitation for 15 minutes.

sWGA

The sWGA reaction was performed in a 96 well plate. The fEach reaction mixture contained 0.50 μL 100x BSA (New England Biolabs), 2 μL 25 mM dNTPs, 1.25μL of each sWGA primer[1] (100 μM), 5 μL 10x Phi29 Reaction buffer (New England Biolabs), 3 μL 1250 U Phi29 Polymerase (New England Biolabs) and 17 μL filtered DNA. The reaction was then placed in a thermocycler programmed to run a stepdown protocol of 5 mins in 35 °C, 10 mins in 34 °C, 15 mins in 33 °C, 20 mins in 32 °C, 30 mins in 31 °C, 16 hours in 30 °C, 15 minutes in 65 °C to inactivate the enzymes and cooling to 4 °C. [1]

References

1. Oyola SO, Ariani CV, Hamilton WL, Kekre M, Amenga-Etego LN, Ghansah A, et al. Whole genome sequencing of Plasmodium falciparum from dried blood spots using selective whole genome amplification. Malar J [Internet]. 2016 Dec 20 [cited 2018 Mar 28];15. Available from: https://www.ncbi.nlm.nih.gov/pmc/articles/PMC5175302/
